# Industry sponsorship and publication bias among animal studies evaluating the effects of statins on atherosclerosis and bone outcomes: a meta-analysis

**DOI:** 10.1186/s12874-015-0008-z

**Published:** 2015-03-06

**Authors:** Andrew T Anglemyer, David Krauth, Lisa Bero

**Affiliations:** Department of Clinical Pharmacy, University of California San Francisco, 50 Beale Street, San Francisco, CA 94143 USA; Charles Perkins Centre, The University of Sydney, NSW Sydney, Australia

**Keywords:** Publication bias, Drug industry, Bias, Meta-analysis, Animal experimentation

## Abstract

**Background:**

The effect that sponsorship has on publication rates or overall effect estimates in animal studies is unclear, though methodological biases are prevalent in animal studies of statins and there may be differences in efficacy estimates between industry and non-industry sponsored studies. In the present analysis, we evaluated the impact of funding source on publication bias in animal studies estimating the effect of statins on atherosclerosis and bone outcomes.

**Methods:**

We conducted two independent systematic reviews and meta-analyses identifying animal studies evaluating the effect of statins on reducing the risk of atherosclerosis outcomes (n = 49) and increasing the likelihood of beneficial bone outcomes (n = 45). After stratifying the included studies within each systematic review by funding source, three separate analyses were employed to assess publication bias in these meta-analyses—funnel plots, Egger’s Linear Regression, and the Trim and Fill methods.

**Results:**

We found potential evidence of publication bias, primarily in non-industry sponsored studies. In all 3 assessments of publication bias, we found evidence of publication bias in non-industry sponsored studies, while in industry-sponsored studies publication bias was not evident in funnel plots and Egger’s regression tests. We also found that inadequate reporting of sponsorship in animal studies is still exceedingly common.

**Conclusions:**

In meta-analyses assessing the effects of statins on atherosclerosis and bone outcomes in animal studies, we found evidence of publication bias, though small numbers of industry-sponsored studies limit the interpretation of the trim-and-fill results. This publication bias is more prominent in non-industry sponsored studies. Industry and non-industry funded researchers may have different incentives for publication. Industry may have a financial interest to publish all preclinical animal studies to maximize the success of subsequent trials in humans, whereas non-industry funded academics may prefer to publish high impact statistically significant results only. Differences in previously published effect estimates between industry- and non-industry sponsored animal studies may be partially explained by publication bias.

**Electronic supplementary material:**

The online version of this article (doi:10.1186/s12874-015-0008-z) contains supplementary material, which is available to authorized users.

## Background

Valid animal research data can generate and test important clinical hypotheses, minimizing the potential risk to patients in clinical trials. In fact, studies of human disease are often developed and improved upon as the result of animal research.

However, prior research suggests that there may be a weak correlation between results in animals studies and subsequent human trials [1-4]. The differences between the results from animal research and human studies may be partially explained by reporting bias in animal studies [5]. Indeed, the publication or lack of publication of empirical findings in research studies that depends on the results’ direction or content is a type of reporting bias referred to as publication bias.

Publication bias in human clinical trials has been measured by comparing publications to meeting abstracts, information on trials approved by human subjects ethics review committees, trial registries, and trials submitted to drug regulatory authorities [6-12]. Publication bias is also estimated using a variety of qualitative and quantitative methods, including funnel plots, Egger regression tests for funnel plot asymmetry, and trim-and-fill methods [13].

To obviate the risk of publication bias in human trials, there are now registries of studies, such as Clinicaltrials.gov which can help reviewers identify all relevant trials. Many journals require that trials be registered before publication. Unlike human studies, however, animal studies do not have a pre-specified registration, though there have been attempts to develop databanks of animal studies to help determine the efficacy of selected interventions. For example, since 2004 animal studies evaluating the efficacy of interventions for stroke have been collated by the Collaborative Approach to Meta-Analysis and Review of Animal Data from Experimental Studies (CAMARADES) [14]. This database has been used in a number of methodological evaluations of animal studies.

Measuring publication bias in animal studies is challenging because not only do few registries of animal studies exist [14] but details about the methods of animal studies are difficult to obtain from animal ethics review committees and regulatory authorities. Publication bias in animal studies is not always considered; Korevaar and colleagues found that a quarter of all meta-analyses of animal studies do not assess publication bias at all [15]. Further, most animal researchers believe that publication biases are highly prevalent and that about only 50% of all animal experiments are published [16], though for-profit researchers, specifically, felt that up to 90% of animal studies are not published [16]. Additionally, Timmer and colleagues found that most abstracts of experimental studies (76%) submitted to a conference were never published, suggesting that non-publication of results may lie with the study’s authors [17].

Though there are methods to estimate publication bias and to quantify the effect that the absence of unpublished data may have on the overall efficacy of a treatment, rarely have investigators been able to estimate the effect of publication bias in meta-analyses of animal research due to the paucity of systematic reviews of animal studies. However, publication bias has previously been identified in animal studies of stroke [18] and neurological disorders [5]. Specifically, based on statistical modeling, Tsilidis and colleagues found that the number of animal studies of neurological disorders with statistically significant positive results far exceeds the expected number of animals studies with positive results [5]. And Sena and colleagues estimated that about one-third of the overall efficacy reported in systematic reviews of stroke in animal studies might be due to publication bias [18]. Further, the researchers estimated that an additional 14% of studies were performed and unpublished [18]. The association of industry funding and bias in human clinical studies has been studied extensively. Lundh and colleagues found that compared to non-industry sponsored human studies, industry-sponsored human studies more often have favorable efficacy results and conclusions which cannot be completely explained by differences in methodology [19].

In the present analysis, we examine whether there is evidence of publication bias in animal studies by expanding the previously performed systematic review and meta-analysis of statins and their effect on atherosclerosis outcomes to include an additional systematic review and meta-analysis of statins and their effect on bone outcomes. As a possible explanation for the larger efficacy estimates observed among non-industry sponsored studies compared with industry-sponsored studies, we hypothesize that publication bias is more evident in non-industry sponsored studies than industry-sponsored studies across various outcomes of interest.

## Methods

### Inclusion/exclusion criteria

We reviewed abstracts of all citations and retrieved studies based on the following inclusion criteria: (1) study conducted in animals (2) original research, defined as a study that presented original data and did not specifically state that it was a review (3) statin drug compared to either a non-statin drug or placebo (4) efficacy outcomes measured (5) assessed effect of statin on at least one clinically relevant atherosclerosis or bone-related outcome (including atherosclerosis--vessel measures, plaque measures, incidence of lesions, measurements of occlusion, plaque type/severity, coronary stenosis, and/or plaque stability; bone—rate formation, bone size, bone density, mechanical strength, bone recovery, and/or bone composition).

Studies with the primary objective of assessing the effect of a combination of a statin and another drug were included if a comparison between a statin-only treatment group and the other drug was made. For further details about inclusion and exclusion criteria, see our previous review [20]. The Institutional Review Board at University of California, San Francisco, approved these reviews.

### Search strategy

We searched Medline from January 1966 to April 2012 using a search term combination developed with input from expert librarians.

See Additional file 1 and previous work [20] for more specific details regarding the search strategy and data collection/extraction (e.g., author characteristics, coding of primary results, and financial ties of primary study authors).

### Sponsorship source

The source of sponsorship for each study was categorized as (1) any industry; (2) fully non-industry; and (3) no sponsorship statement.

### Statistical analysis-meta analyses

The meta-analytical statistical methods employed for both reviews have been described in our previous publication [21]. In brief, for the meta-analysis, we extracted data for mean outcome, standard deviation (SD) or standard error (SE), and the number of treated and untreated animals to test our hypothesis that the effect sizes of atherosclerosis and bone outcomes are affected by industry sponsorship and/or increased risks of bias. We calculated the effect of statins using a standardized mean difference (SMD) for each outcome and we pooled the data across studies using random-effects models [22] for each type of study—studies of statin use on reducing atherosclerosis harms outcomes, and studies of statin use on improving beneficial bone outcomes. The SMD null hypothesis (Ho: estimate = 0) states that there is no difference in effect of statin-use on risk of atherosclerosis harms outcomes or likelihood for beneficial bone outcomes when compared to a control/placebo. A number less than zero suggests that the statin reduces the risk of atherosclerosis harms outcomes or reduces the likelihood of beneficial bone outcomes when compared to control or placebo. A number greater than zero suggests that the statin increases the risk of atherosclerosis harms outcomes or increases the likelihood of beneficial bone outcomes when compared to the control or placebo.

### Statistical analysis-publication bias assessments

Three separate analyses were employed to assess publication bias—funnel plots, Egger’s Linear Regression, and the Trim and Fill methods. The effects of statins on atherosclerosis outcomes and bones outcomes were examined separately because of the heterogeneity of the outcomes and the direction of beneficial effect. We stratified the pooled effect estimates by declared sponsorship sources to evaluate differences in risks of publication bias by funding source.

#### Funnel plots

A qualitative method for analyzing publication bias is the funnel plot. The x-axis in the present analysis is the treatment effect (SMD) and the y-axis is the standard error of that treatment effect. In the absence of bias, a funnel plot should be a symmetrical inverted funnel. In the presence of bias, smaller studies with no beneficial effects would be missing, thus creating an asymmetrical funnel. Asymmetry in a funnel plot suggests that there is a systematic difference between larger and smaller studies and/or that there is publication bias.

#### Egger’s linear regression

Funnel plot asymmetry can be tested using Egger’s linear regression method. The standardized treatment effect is regressed on the precision—with the weight equal to the precision, a weighted regression of the treatment effect size on its standard error is calculated. In the absence of bias, the bias coefficient would be close to zero and the regression line would have no slope. In the presence of bias, bias coefficients will be significantly different than zero and the regression line will have a slope.

#### Trim and fill

We also used the trim and fill method of assessing the potential effect missing studies may have had on our observed results. After estimating the number of studies in the outlying side of a funnel plot assumed to be affected by publication bias, the method trims off the “asymmetric” side and uses the symmetric remaining studies to estimate the true center of the funnel plot. Lastly, the trimmed studies are replaced with their missing “counterparts” around the center. While this method should not be viewed as an adjustment for publication bias, it allows for estimating the potential impact of publication bias on pooled estimates.

Statistical analyses were conducted using R packages rmeta, meta, and metafor.

We conducted a sensitivity analysis by categorizing studies with no disclosed sponsorship as industry sponsored. We based this recategorization on the assumption that funding for industry sponsored studies is less likely to be disclosed than funding from other sources such as government as these other sources require acknowledgement of their funding.

## Results

### Summary effects

Using random-effects models, we conducted two independent systematic reviews and meta-analyses identifying animal studies evaluating the effect of statins on reducing the risk of atherosclerosis outcomes and increasing the likelihood of beneficial bone outcomes. In the atherosclerosis studies, efficacy was defined as a decrease in a measure of atherosclerosis. We identified 49 unique studies evaluating 184 atherosclerosis outcomes in 3498 animals and the pooled effect was SMD = −1.25 (95% CI −1.56, −0.94) with substantial heterogeneity (I^2^ = 73%) (Table 1). A large proportion of these studies was funded by non-industry sources (n = 23), while industry sources funded 15 studies and 11 studies had no funding statement. In the bone studies, efficacy was defined as an increased in beneficial bone outcomes. We identified 45 unique studies evaluating 654 beneficial bone outcomes in 1986 animals and the pooled effect was SMD = 0.42 (95% CI 0.00-0.83) with substantial heterogeneity (I^2^ = 89%). The majority of studies of bone outcomes were by non-industry sources (n = 28), and only 6 studies were funded by industry sources, while 11 studies failed to disclose a funding source.

**Table 1:** **Effect size by outcome and funding source**

**Outcome**	**Number of studies**	**Number of animals**	**Heterogeneity (I** ^**2**^ **)**	Effect size* (95% CI)
Atherosclerosis	49	3498	73	−1.25 (−1.56, −0.94)
Funding Source				
Industry	15		18	−0.73 (−1.00, −0.47)
Non-industry	23		84	−1.99 (−2.68, −1.31)
No Statement	11		0	−0.93 (−1.24, −0.61)
Bone	45	1986	89	0.42 (0.00, 0.83)
Funding Source				
Industry	6		60	0.13 (−0.48, 0.73)
Non-industry	28		91	0.48 (−0.10, 1.06)
No Statement	11		80	0.39 (−0.36, 1.14)

*Standardized Mean Difference As Estimated in DerSimonian Laird Random-Effects Models.

Industry sponsored studies had smaller efficacy estimates than non-industry sponsored studies (Table 1). The efficacy of statins on reducing atherosclerosis (e.g., summary effect sizes) among industry-sponsored atherosclerosis studies (−0.73; 95% CI −1.00, −0.47) was significantly less than the efficacy estimates among non-industry sponsored studies (−1.99; 95% CI −2.68, −1.31) (test for subgroup differences p value < 0.001). There was no significant difference in beneficial bone outcomes between industry sponsored (0.13; 95% CI −0.48, 0.73) and non-industry sponsored studies (0.48; 95% CI −0.10, 1.06) (p value = 0.41).

### Publication bias assessments

#### Funnel plots

**Figure 1:**
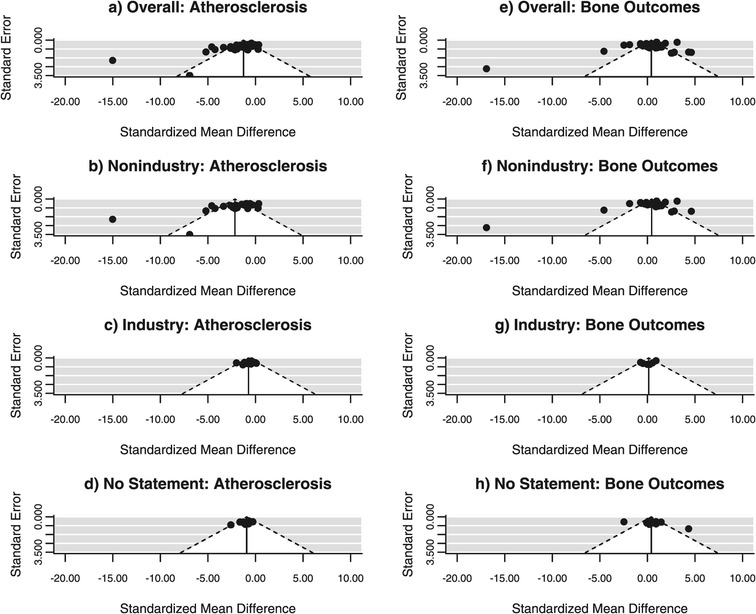
**Funnel plots.** Data from meta-analyses of atherosclerosis studies **(a-d)** and bone studies **(e-h)**. Funnel plots show standard error plotted against standardized mean difference with diagonal lines showing the expected 95% confidence intervals around the summary estimate. In the absence of heterogeneity, 95% of studies should lie within the diagonal lines.

Across all studies, (Figure 1; panels a, e) there appears to be publication bias in both atherosclerosis and bone studies as assessed by funnel plot asymmetry. After stratification by funding source, bias appears to remain in atherosclerosis and bone studies with no industry sponsorship (Figure 1; panels b, f), though bias is not as apparent in studies with industry sponsorship (Figure 1; panels c, g).

#### Egger’s linear regression

**Figure 2:**
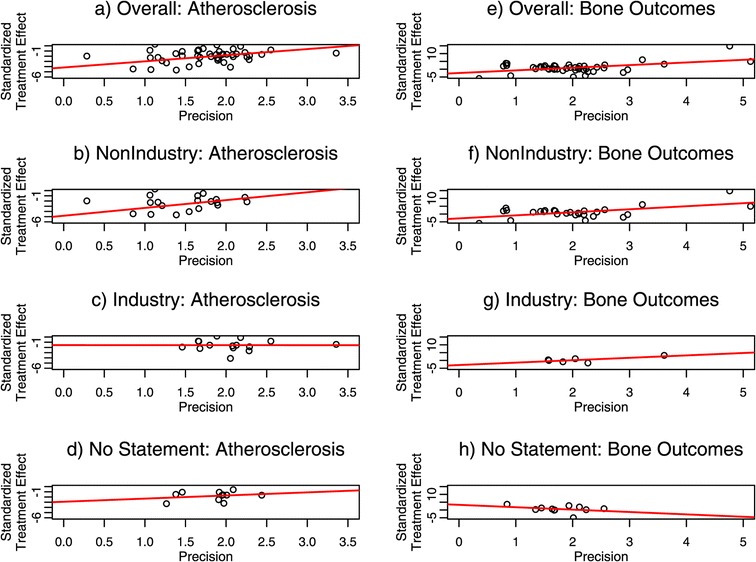
**Egger’s linear regression method.** Data from meta-analyses of atherosclerosis studies **(a-d)** and bone studies **(e-h)**. Plots show the standardized treatment effect plotted against precision (inverse of standard error). In the absence of funnel plot asymmetry, the slope of the regression line will be zero.

Funnel plot asymmetry was tested using Egger’s linear regression. Across all studies, (Figure 2; panels a, e) there appears to be bias in both atherosclerosis and bone studies. For atherosclerosis studies, the bias coefficient is −4.17 (95% CI −5.61, −2.73; p value < 0.0001), while for bone studies the bias coefficient is −2.43 (95% CI −4.42, −0.43; p value = 0.021). After stratification by funding source, bias (i.e., funnel plot asymmetry) appears to remain in atherosclerosis and bone studies with no industry sponsorship (Figure 2; panels b, f)—bias coefficients: −4.83 (95% CI −7.25, −2.41; p value < 0.0001), −2.80 (95% CI −5.42, −0.17; p value = 0.043), respectively. However, bias is absent in industry-sponsored studies of atherosclerosis and bone outcomes (bias coefficients: −1.56; 95% CI −4.26, 1.15; p value = 0.270. -2.95; 95% CI −6.60, 0.69; p value = 0.180, respectively) (Figure 2; panels c, g). Similarly, bias is absent in studies with no statement of sponsorship (−2.91; 95% CI −5.96, 0.13; p value = 0.088. 3.30; 95% CI −2.11, 8.71; p value = 0.253, in atherosclerosis and bone studies, respectively) (Figure 2; panels d, h).

#### Trim and fill

**Figure 3:**
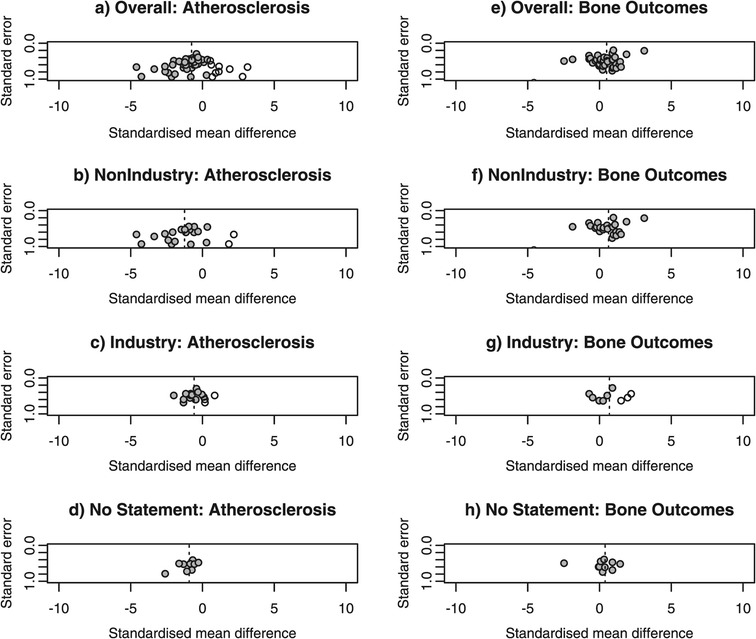
**Trim and fill method.** Data from meta-analyses of atherosclerosis studies **(a-d)** and bone studies **(e-h)**. Funnel plots show the standard error plotted against the standardized mean difference. The imputed missing studies are shown as open circles on the plots.

Across all studies, the pooled effect of statins on atherosclerosis outcomes is −1.25 (95% CI −1.56, −0.94). After trimming and imputing 12 studies, the pooled estimate is smaller (SMD = −0.76; 95% CI −1.13, −0.39), which suggests that in the absence of publication bias the summary estimate may have been approximately 40% lower than the estimate we obtained (Figure 3; panel a). Overall, the 12 “missing” studies accounted for an additional 24% more studies than those identified in our systematic review. This is an estimate for the percentage of conducted animal studies that were unreported or not identified in our review. After stratification by funding source, the pooled effect of statins on atherosclerosis outcomes in studies without industry sponsorship is −1.99 (95% CI −2.68, −1.31) and the pooled effect after the trim and fill method (5 studies) is reduced (−1.25; 95% CI −2.05, −0.45) (Figure 3; panel b), a summary effect approximately 40% lower than the summary estimate obtained in the meta-analysis. The 5 “missing” studies accounted for an additional 22% of animal studies without industry sponsorship that were unreported. The pooled effect of statins on atherosclerosis outcomes in studies with industry sponsorship is −0.73 (95% CI −1.00, −0.47) and the pooled effect after the trim and fill method (3 studies) is reduced (−0.58; 95% CI −0.89, −0.28) (Figure 3; panel c), a summary effect approximately 20% lower than the summary estimate obtained in the meta-analysis. The 3 “missing” studies accounted for an additional 20% of animal studies with industry sponsorship that were unreported. The pooled effect of statins on atherosclerosis outcomes in studies with no sponsorship statement is −0.93 (95% CI −1.24, −0.61) and the trim and fill method identified no “missing” studies (Figure 3; panel d), suggesting no publication bias present.

Similarly, across all studies, the pooled effect of statins on bone outcomes is 0.42 (95% CI 0.00, 0.83). After trimming and imputing 1 study, the pooled estimate is slightly increased (SMD = 0.50; 95% CI 0.06, 0.93) (Figure 3; panel e), an estimate approximately 16% greater than the summary estimate obtained in the meta-analysis. The 1 “missing” study accounted for an additional 2% of conducted animal studies that were unreported. After stratification by funding source, the pooled effect of statins on bone outcomes in studies without industry sponsorship is 0.48 (95% CI −0.10, 1.06) and the pooled effect after the trim and fill method (1 study) is increased (0.63; 95% CI 0.02, 1.24), an estimate approximately 30% greater than the summary estimate obtained in the meta-analysis (Figure 3; panel f). The 1 “missing” studies accounted for an additional 4% of conducted animal studies that were without industry sponsorship that were unreported. The pooled effect of statins on bone outcomes in studies with industry sponsorship is 0.13 (95% CI −0.48, 0.73) and the pooled effect after the trim and fill method (3 studies) is substantially changed (0.69; 95% CI 0.02, 1.37) (Figure 3; panel g). The 3 “missing” studies accounted for an additional 50% of conducted animal studies that were sponsored by industry and were unreported. The pooled effect of statins on bone outcomes in studies with no statement of industry sponsorship is 0.39 (95% -0.36, 1.14) and the trim and fill method identified 0 “missing” studies (Figure 3; panel h).

### Sensitivity analysis

To estimate the effect of non-disclosure of sponsorship on our results, we performed all analyses after re-categorizing each study without a sponsorship statement as an industry-sponsored study (see Additional files 2, 3, 4 and 5). Differences between industry and non-industry studies are less pronounced, particularly for estimates from Egger’s regression tests. Specifically, publication bias remains absent in bone studies with industry sponsorship (Additional file 4: Figure S2; panel f)—bias coefficient: 0.24 (95% CI −3.19, 3.67; p value = 0.891). However, in atherosclerosis studies with industry sponsorship, publication bias is present −2.13 (95% CI −4.01, −0.25; p value = 0.033) (Additional file 4: Figure S2; panel c), suggesting that assuming that studies without sponsorship statements are industry-sponsored can have an important impact on the estimates.

## Discussion

In meta-analyses assessing the effects of statins on atherosclerosis and bone outcomes in animal studies, we found potential evidence of publication bias. Further, this publication bias is more prominent in non-industry sponsored studies. Using 3 different estimates of publication bias, we found evidence of publication bias in non-industry sponsored studies, though in industry-sponsored studies publication bias was only evident in funnel plots and Egger’s regression tests. These findings support our hypothesis that publication bias is more evident in non-industry sponsored studies than industry sponsored studies. Thus, the greater efficacy estimates observed in meta-analyses of non-industry sponsored animal studies compared to industry sponsored studies [21] may be due to the failure to publish negative non-industry sponsored studies.

In this study, the meta-analyses of both atherosclerosis and bone outcomes show that the effects of statins measured in non-industry sponsored studies yielded greater efficacy than industry-sponsored studies, while displaying signs of publication bias. However, results from atherosclerosis and bone outcomes from industry-sponsored studies, while yielding smaller effects, displayed fewer signs of publication bias. Though trim-and-fill methods in industry-sponsored studies of bone outcomes suggest the possibility of publication bias, there were only 6 identified industry-sponsored studies, making the trim-and-fill results difficult to interpret. In sensitivity analyses in which the studies without a statement of financial support were re-categorized as industry-sponsored, publication bias was absent in industry-sponsored studies of bone outcomes.

Publication bias is one possible reason for observed, perceived industry bias [19]. The extent that sponsorship influences overall effect estimates in animal studies is unclear. We previously found that the effect of statins on atherosclerosis efficacy outcomes was significantly larger for animal studies sponsored by non-industry sources versus studies sponsored by industry [21], but a meta-analyses of the effects of thiazolidiones on glucose reduction in animals had conflicting results [23]. Further investigation of funding bias in larger cohorts of animal studies is needed. Substantial methodological risks of bias existed across all studies in our animal sample, although we could not detect differences in risks of bias between the non-industry and industry sponsored studies. Therefore, publication bias is a possible explanation for the larger effect sizes observed in the non-industry sponsored animal studies compared to the industry-sponsored studies.

Though rarely studied, publication bias in animal studies has been noted in previous analyses [5,18]. Overall, animal studies with statistically significant results are much more likely to get published than studies with neutral or statistically non-significant results [15,16,24]. Sena and colleagues studied publication bias in animal studies and estimated that due to “missing” or unpublished studies the efficacy reported in 525 publications was overestimated by approximately 31% [18].

Reasons for differences in the extent of publication bias in industry and non-industry funded studies are not clear. Though we found little evidence of publication bias in industry-sponsored studies, ter Riet and colleagues surveyed laboratory animal researchers in for-profit settings and they estimated that only about 10% of their animal research ever gets published, while non-industry sponsored researchers report that 80% of their work is published [16]. This study speculated that potential causes of the low publication rate among for-profit animal researchers could include opinions of peer reviewers and no statistically significant findings [16]. Conversely, industry and non-industry funded researchers may have different incentives for publication. Industry may have a financial interest to publish all preclinical animal studies to maximize the success of subsequent trials in humans, whereas non-industry funded academics may prefer to publish high impact statistically significant results only [25].

Studies of publication bias in human research have suggested additional factors that may be associated with a lack of publication. Vulnerability to publication bias in human studies is more prominent in specific research areas and authorship regions [26]. In fact, there is evidence that industry-sponsored and US-based studies are more likely to be published than non-industry sponsored or non-US based studies [27]. Furthermore, high quality studies and studies where the publishing journal is located in the same country as the corresponding author are more likely to be published [10]. It is believed that any regional difference may be partially explained by the assumption that researchers in some regions are often faced with greater expectations to publish, while results from researchers in other regions are usually only published if considered overtly impactful [26]. And, editorial boards of English language journals are mainly comprised of Americans, which could also lead to a bias in favor of US research [27,28]. Though it has been suggested that US researchers may report more positive and statistically significant results [29], others have not found similar results [27].

There have been recent efforts to improve reporting standards for animal research, and we have shown that reporting of randomization, accounting for all animals, and sample size have improved slightly since the publication of the ARRIVE guidelines [21]. However, guidelines for reporting animal research have focused on reporting risk of bias and other characteristics of the studies, rather than ensuring that entire studies and full outcomes are reported [30,31]. While medical journals have taken steps to improve mandatory reporting and follow-up of protocols in human studies by requiring registration of studies before publication [32], the same requirements have not yet been established for laboratory animal research. Prospective registration of animal studies, release of information on animal studies that have received ethical approval, and having funders require publication of the results of animal studies are other possible ways to reduce publication bias in animal research [30,31]. Further, through registration and publication of animal studies, future human volunteers may have less unnecessary enrollment in trials [33].

This study of publication bias should be interpreted with an understanding of the inherent limitations. First, though we performed an exhaustive search and systematic review of animal studies and their effects on atherosclerosis and bone outcomes, we may have missed some relevant studies. The trim-and-fill results indicate that there are “missing” industry and non-industry sponsored studies, suggesting publication bias. However, these “missing” studies could also be studies that were not identified in our search. Second, we may have lacked reasonable dispersion of the sample size to get a robust estimate of publication bias. In fact, due to a small sample size of industry-sponsored studies evaluating bone outcomes, the results that failed to indicate publication bias in this subgroup, in particular, may have occurred as a result of Type II error. However, in the sensitivity analysis in which we re-categorized studies with no funding statement as industry sponsored, Egger’s regression test for funnel plot asymmetry remained non-significant (p value = 0.891). Third, the impression of publication bias as illustrated in the funnel plots may be an artifact of high precision studies truly having a different effect size. However, the funnel plots, if considered together with Egger’s regression test and the trim-and-fill methods remain suggestive of publication bias. And while the Cochrane Handbook recommends a minimum of 10 studies to be included in any publication bias analysis [34], we acknowledge that with a larger sample size of studies identified in each subgroup (particularly industry-sponsored studies of bone outcomes) we would have likely gained more insight regarding the presence of publication bias. Fourth, in the systematic reviews, many of the studies we included had small samples sizes and measured multiple outcomes in a single animal. Additionally, the data we evaluated included disparate outcomes, durations of follow-up, different species, and different types of statins. All of these factors could contribute to the overall heterogeneity in the results; the impacts these differences have on publication bias is unknown. Further details and our methods for dealing with these issues are previously described [21]. Lastly, our results are only generalizable to the research questions specifically reviewed in the two systematic reviews (e.g., bone outcomes and atherosclerosis). Though publication bias may be prominent in multiple areas of animal research, the direction and strength of this bias is unpredictable based on our present results.

## Conclusions

We employed multiple methods used to assess publication bias and our analyses suggest that there is evidence of publication bias in non-industry sponsored studies, while in industry-sponsored studies publication bias is not as evident. Furthermore, we note that inadequate reporting of sponsorship in animal studies is very common. Differences in results between industry- and non-industry sponsored animal studies may be partially explained by publication bias.

## Additional files

Additional file 1:
**Search Strategies.**
Additional file 2: Table S1. Summary of Collected Data (Funding Source Combined). Additional file 3: Figure S1. Funnel Plots (Funding Source Combined). Legend: Data from meta-analyses of atherosclerosis studies (a-c) and bone studies (d-f). Funnel plots show standard error plotted against standardized mean difference with diagonal lines showing the expected 95% confidence intervals around the summary estimate. In the absence of heterogeneity, 95% of studies should lie within the diagonal lines. Additional file 4: Figure S2. Egger’s Linear Regression Method (Funding Source Combined). Legend: Egger’s Linear Regression Method. Data from meta-analyses of atherosclerosis studies (a-c) and bone studies (d-f). Plots show the standardized treatment effect plotted against precision (inverse of standard error). In the absence of funnel plot asymmetry, the slope of the regression line will be zero. Additional file 5: Figure S3. Trim and Fill Method (Funding Source Combined). Legend: Trim and Fill Method. Data from meta-analyses of atherosclerosis studies (a-c) and bone studies (d-f). Funnel plots show the standard error plotted against the standardized mean difference. Additional missing studies that were imputed using this method are open circles on the plots.

## Abbreviations

SE: Standard error
SD: Standard deviation
SMD: Standardized mean difference
CI: Confidence interval
