# Supplementary material for: Industry sponsorship and publication bias among animal studies evaluating the effects of statins on atherosclerosis and bone outcomes: a meta-analysis
Source: BMC Med Res Methodol. 2015 Mar 6;15:12. doi: 10.1186/s12874-015-0008-z (PMC4353470; doi:10.1186/s12874-015-0008-z)
Supplement: Additional file 1: — Search Strategies. [file 12874_2015_8_MOESM1_ESM.docx]

Additional Text 1

Our search strategy contained the following MeSH terms, text words and word variants:

(atorvastatin OR cerivastatin OR fluvastatin OR lovastatin OR mevastatin OR pitavastatin OR pravastatin OR rosuvastatin OR simvastatin OR hydroxymethylglutaryl-CoA reductase inhibitors) AND (animal* OR preclinical OR "pre-clinical" OR mice OR rats OR rabbits OR dog OR dogs OR monkey OR monkeys OR "animal experimentation"[MeSH Terms] OR "models, animal"[MeSH Terms] OR "invertebrates"[MeSH Terms] OR "Animals"[MH] OR "animal population groups"[MeSH Terms]) NOT (humans[mh] NOT animals[mh:noexp]) AND (health effect OR health effects OR toxic OR toxicity OR toxicities OR efficacy OR efficacies OR toxicology OR safety OR harm* OR drug effects[sh] OR therapeutic use[sh:noexp] OR adverse effects[sh] OR poisoning[sh] OR pharmacology[sh:noexp] OR chemically induced[sh]) AND eng[la] NOT review[pt] NOT systematic review* NOT meta-analysis[pt]
