# Supplementary material for: Industry sponsorship and publication bias among animal studies evaluating the effects of statins on atherosclerosis and bone outcomes: a meta-analysis
Source: BMC Med Res Methodol. 2015 Mar 6;15:12. doi: 10.1186/s12874-015-0008-z (PMC4353470; doi:10.1186/s12874-015-0008-z)
Supplement: Additional file 2: Table S1. — Summary of Collected Data (Funding Source Combined). [file 12874_2015_8_MOESM2_ESM.docx]

Additional Table S1

Table S1: Summary of Collected Data (Funding Source Combined)

| Outcome | Number of Studies | Number of Animals | Heterogeneity (I^2^) | Effect Size* (95% CI) |
| --- | --- | --- | --- | --- |
| Atherosclerosis | 49 | 3498 | 73 | -1.25 (-1.56, -0.94) |
| Funding Source |  |  |  |  |
| Industry  Nonindustry | 15  34 |  | 11  84 | -0.81 (-1.01, -0.60)  -1.99 (-2.68, -1.31) |
| Bone | 45 | 1986 | 89 | 0.42 (0.00, 0.83) |
| Funding Source |  |  |  |  |
| Industry  Nonindustry | 17  28 |  | 75  91 | 0.26 (-0.24, 0.75)  0.48 (-0.10, 1.06) |

*Standardized Mean Difference As Estimated in DerSimonian Laird Random-Effects Models
