# Supplementary material for: Industry sponsorship and publication bias among animal studies evaluating the effects of statins on atherosclerosis and bone outcomes: a meta-analysis
Source: BMC Med Res Methodol. 2015 Mar 6;15:12. doi: 10.1186/s12874-015-0008-z (PMC4353470; doi:10.1186/s12874-015-0008-z)
Supplement: Additional file 3: Figure S1. — Funnel Plots (Funding Source Combined). Legend: Data from meta-analyses of atherosclerosis studies (a-c) and bone studies (d-f). Funnel plots show standard error plotted against standardized mean difference with diagonal lines showing the expected 95% confidence intervals around the summary estimate. In the absence of heterogeneity, 95% of studies should lie within the diagonal lines. [file 12874_2015_8_MOESM3_ESM.docx]

Additional Figure S1

Figure S1

Funnel plots. Data from meta-analyses of atherosclerosis studies (a-c) and bone studies (d-f). Funnel plots show standard error plotted against standardized mean difference with diagonal lines showing the expected 95% confidence intervals around the summary estimate. In the absence of heterogeneity, 95% of studies should lie within the diagonal lines.
