# Supplementary material for: Industry sponsorship and publication bias among animal studies evaluating the effects of statins on atherosclerosis and bone outcomes: a meta-analysis
Source: BMC Med Res Methodol. 2015 Mar 6;15:12. doi: 10.1186/s12874-015-0008-z (PMC4353470; doi:10.1186/s12874-015-0008-z)
Supplement: Additional file 4: Figure S2. — Egger’s Linear Regression Method (Funding Source Combined). Legend: Egger’s Linear Regression Method. Data from meta-analyses of atherosclerosis studies (a-c) and bone studies (d-f). Plots show the standardized treatment effect plotted against precision (inverse of standard error). In the absence of funnel plot asymmetry, the slope of the regression line will be zero. [file 12874_2015_8_MOESM4_ESM.docx]

Additional Figure S2

Figure S2


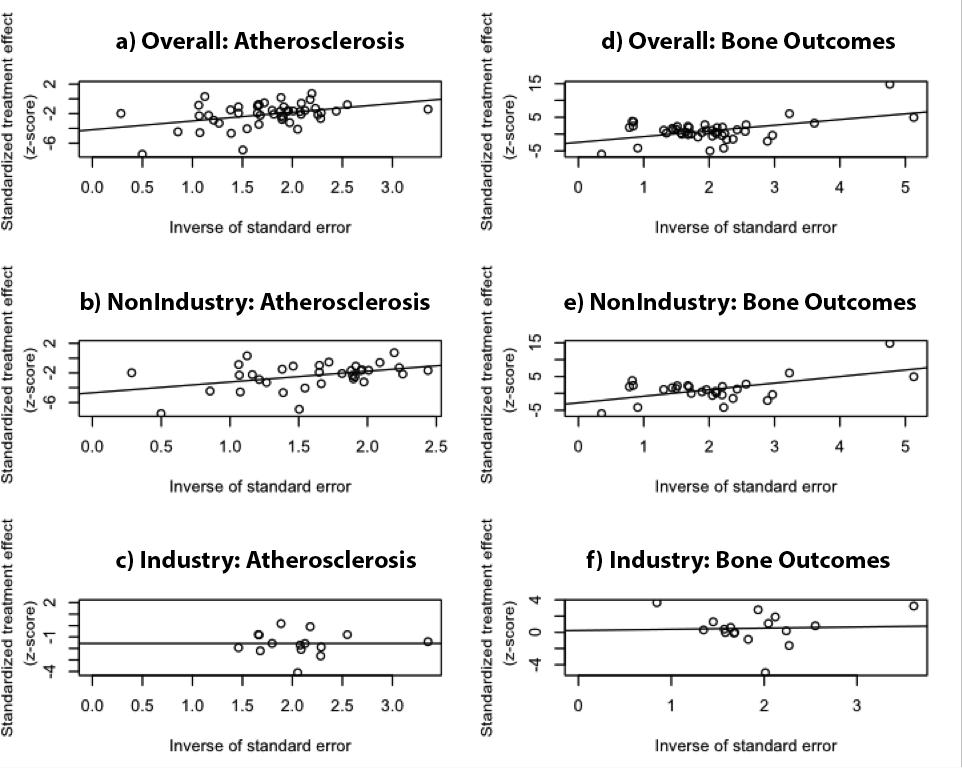


Egger’s Linear Regression Method. Data from meta-analyses of atherosclerosis studies (a-c) and bone studies (d-f). Plots show the standardized treatment effect plotted against precision (inverse of standard error). In the absence of funnel plot asymmetry, the slope of the regression line will be zero.
