# Supplementary material for: Industry sponsorship and publication bias among animal studies evaluating the effects of statins on atherosclerosis and bone outcomes: a meta-analysis
Source: BMC Med Res Methodol. 2015 Mar 6;15:12. doi: 10.1186/s12874-015-0008-z (PMC4353470; doi:10.1186/s12874-015-0008-z)
Supplement: Additional file 5: Figure S3. — Trim and Fill Method (Funding Source Combined). Legend: Trim and Fill Method. Data from meta-analyses of atherosclerosis studies (a-c) and bone studies (d-f). Funnel plots show the standard error plotted against the standardized mean difference. Additional missing studies that were imputed using this method are open circles on the plots. [file 12874_2015_8_MOESM5_ESM.docx]

Additional Figure S3

Figure S3

Trim and Fill Method. Data from meta-analyses of atherosclerosis studies (a-c) and bone studies (d-f). Funnel plots show the standard error plotted against the standardized mean difference. Additional missing studies that were imputed using this method are open circles on the plots.
